# Use of Lipid Nanocarriers to Improve Oral Delivery of Vitamins

**DOI:** 10.3390/nu11010068

**Published:** 2019-01-01

**Authors:** Ching-Yun Hsu, Pei-Wen Wang, Ahmed Alalaiwe, Zih-Chan Lin, Jia-You Fang

**Affiliations:** 1 Department of Nutrition and Health Sciences, Chang Gung University of Science and Technology, Kweishan, Taoyuan 33302, Taiwan; cyhsu@mail.cgust.edu.tw; 2 Research Center for Food and Cosmetic Safety and Research Center for Chinese Herbal Medicine, Chang Gung University of Science and Technology, Kweishan, Taoyuan 33302, Taiwan; 3 Department of Medical Research, China Medical University Hospital, China Medical University, Taichung 40402, Taiwan; pan@mail.cgu.edu.tw; 4 Department of Pharmaceutics, College of Pharmacy, Prince Sattam Bin Abdulaziz University, Al Kharj 11942, Saudi Arabia; alalaiwe@gmail.com; 5 Graduate Institute of Biomedical Sciences, Chang Gung University, Kweishan, Taoyuan 33302, Taiwan; jane2707@kimo.com; 6 Pharmaceutics Laboratory, Graduate Institute of Natural Products, Chang Gung University, Kweishan, Taoyuan 33302, Taiwan; 7 Chinese Herbal Medicine Research Team, Healthy Aging Research Center, Chang Gung University, Kweishan, Taoyuan 33302, Taiwan; 8 Department of Anesthesiology, Chang Gung Memorial Hospital, Kweishan, Taoyuan 33302, Taiwan

**Keywords:** lipid nanocarrier, vitamin, nutrient, oral absorption, bioavailability

## Abstract

The chemical environment and enzymes in the gastrointestinal (GI) membrane limit the oral absorption of some vitamins. The GI epithelium also contributes to the poor permeability of numerous antioxidant agents. Thus, lipophilic vitamins do not readily dissolve in the GI tract, and therefore they have low bioavailability. Nanomedicine has the potential to improve the delivery efficiency of oral vitamins. In particular, the use of lipid nanocarriers for certain vitamins that are administered orally can provide improved solubility, chemical stability, epithelium permeability and bioavailability, half-life, nidus targeting, and fewer adverse effects. These lipid nanocarriers include self-emulsifying drug delivery systems (SEDDSs), nanoemulsions, microemulsions, solid lipid nanoparticles (SLNs), and nanostructured lipid carriers (NLCs). The use of nontoxic excipients and sophisticated material engineering of lipid nanosystems allows for control of the physicochemical properties of the nanoparticles and improved GI permeation via mucosal or lymphatic transport. In this review, we highlight recent progress in the development of lipid nanocarriers for vitamin delivery. In addition, the same lipid nanocarriers used for vitamins may also be effective as carriers of vitamin derivatives, and therefore enhance their oral bioavailability. One example is the incorporation of d-α-tocopheryl polyethylene glycol succinate (TPGS) as the emulsifier in lipid nanocarriers to increase the solubility and inhibit P-glycoprotein (P-gp) efflux. We also survey the concepts and discuss the mechanisms of nanomedical techniques that are used to develop vitamin-loaded nanocarriers.

## 1. Introduction

Oral administration is often the best route for bioactive agents that have therapeutic or preventive effects in which patient compliance is important. However, oral bioactive agents experience the harsh environment of the gastrointestinal (GI) tract, which can reduce their solubility, stability, and absorption [1]. In particular, chemical and enzymatic degradation reduces the amount that is available for absorption, the GI membrane prevents permeation into the systemic circulation, and molecules with low water solubility do not readily dissolve in the GI tract. All of these factors contribute to the low bioavailability and bioactivity of oral drugs [2].

Bioavailability, which is a major pharmacokinetic parameter, is defined as the extent and rate to which an active compound is absorbed from a product and becomes available at the target site. An individual must have adequate uptake of bioactive compounds, such as vitamins and nutrients, for these agents to provide beneficial effects. Thus, previous researchers have developed structurally sophisticated formulations to improve physical stability, protect the active ingredients from degradation, and provide controlled release during digestion to improve absorption and bioavailability [3]. The use of prodrugs, permeation enhancers, solid dispersion, polymeric complexes, and molecular encapsulation can improve oral bioavailability [4]. Colloidal delivery systems, consisting of microsized or nanosized encapsulation formulations, can also improve the absorption and bioavailability of oral bioactive agents. In particular, drug formulations in the nanometer range have better pharmacokinetics than those in the micrometer range [5], because smaller carriers have a greater effective surface area. This greater surface area increases the dissolution rate and bioavailability of the active agents.

Nanoformulations can also facilitate enterocyte uptake, and thereby increase absorption. There is evidence that nanocarriers form a protective shield that maintains their stability in the GI tract [6], so that the dose can be reduced. The surface properties of nanoparticles modulate and control the release and absorption rate. Furthermore, nanocarriers can function as vehicles for delivery of a variety of chemicals, such as small-molecule drugs, antioxidants, nutrients, vitamins, peptides, proteins, antibodies, and RNAs [7]. Some nanocarriers were designed to increase oral bioavailability, such as nanocrystals, polymeric nanoparticles, dendrimers, silica nanoparticles, nanotubes, liposomes, and lipid-based nanoparticles.

**Figure 1:**
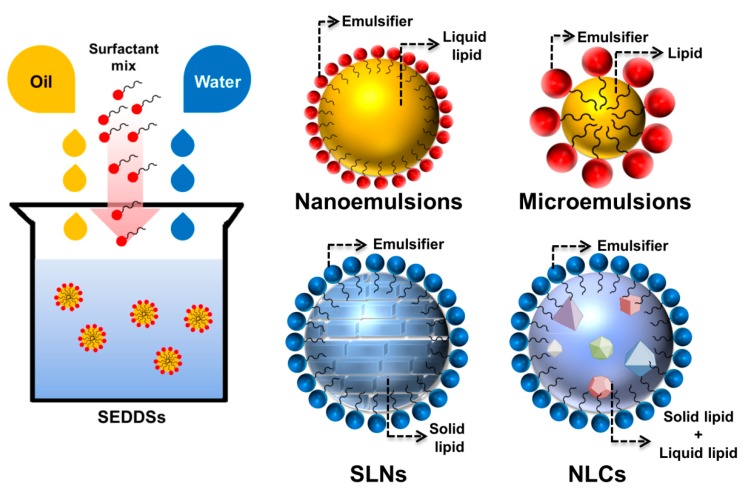
Structures of lipid-based nanoparticles: self-emulsifying drug delivery systems (SEDDSs), nanoemulsions, microemulsions, solid lipid nanoparticles (SLNs), and nanostructured lipid carriers (NLCs).

Use of lipid-based nanocarriers for the oral administration of bioactive agents with poor water solubility can overcome the problems of low bioavailability. The excipients in the lipid nanoparticles, such as emulsifiers or surfactants, can also improve bioavailability [8]. Lipid-based nanodelivery systems, such as self-emulsifying drug delivery systems (SEDDSs), nanoemulsions, microemulsions, solid lipid nanoparticles (SLNs), and nanostructured lipid carriers (NLCs), encapsulate bioactive compounds and increase their solubility and bioavailability when administered by the oral route (Figure 1). Lipid nanoparticles have the characteristics of nanosized particles and lipid solubility, leading to improved pharmacokinetic parameters and biocompatibility, reduced toxicity, and facilitating scale-up for industrial production [9]. The human body also easily takes up lipid-based nanocarriers. Their digestion involves the dispersion of fat globules into an emulsion with high surface area, enzymatic hydrolysis of the triglyceride lipid at the lipid-water interface, and the dispersion of the bioactive product into the absorbed form [10]. The encapsulation of drugs or bioactive agents by the use of lipid-based nanosystems can also reduce the influence of food intake on absorption and variability among subjects, due to their controlled release.

**Figure 2:**
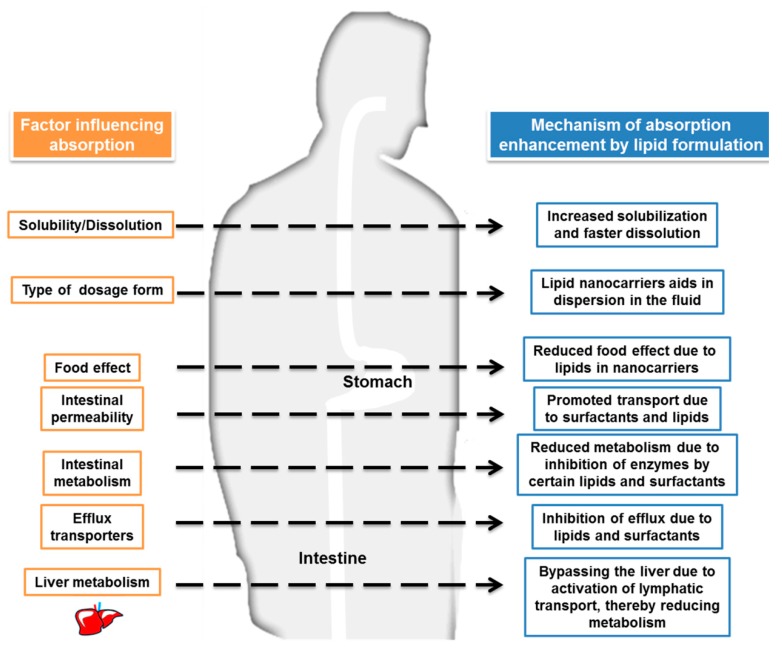
Possible mechanisms for enhancement of vitamin bioavailability using lipid-based delivery systems.

Lipid nanoparticles can improve the bioavailability of orally administered bioactive agents via various mechanisms. First, lipid nanoparticles have enhanced solubilization and rapid dissolution because of lipid inclusion within lipid nanoparticles. The administration of lipids can activate biliary and pancreatic secretion, and these assist in lipid digestion [11]. The adhesiveness of lipid nanosystems prolongs the presence of bioactive molecules in the GI tract, and this increases their absorption. For example, Peyer’s patches target gut-associated lymphoid tissue and absorption by M cells, allowing lipid nanoparticle delivery. Lipids that have high solubility in triglycerides undergo lymphatic transport, leading to increased bioavailability because they are not subjected to first-pass metabolism [12]. The ability of lipids and surfactants to open tight junctions in the intestine contributes to their enhanced permeability; their ability to inhibit P-glycoprotein (P-gp) efflux from the intestinal epithelium also increases intestinal permeability [13]. In a molecular or cellular level, previous studies [14,15,16,17] have shown a facile uptake of lipid-based nanoparticles into gastric and intestinal epithelium cells. The transport of lipid nanoparticles into epithelium cells can involve in various pathways, including lipid raft-dependent endocytosis, clathrin-mediated endocytosis, and macropinocytosis [18]. Thus, lipid-based delivery systems can increase the bioavailability of oral bioactive agents by numerous mechanisms (Figure 2).

Lipid-based nanoformulations typically consist of lipids, emulsifiers/coemulsifiers, and hydrophilic solvents. Water is the most common hydrophilic solvent. The chemical properties of the individual ingredients and their proportions affect molecular entrapment, solubilization, stability, and oral absorption. Thus, different types of lipids can be formulated into lipid nanocarriers from long, medium, and short triglycerides (LCTs, MCTs, and SCTs) [19]. LCTs are digested more slowly than MCTs and SCTs, suggesting that lipase activity declines with chain length. In addition, bioactive agents are generally better maintained in a solubilized state in LCTs and MCTs than in SCTs. Due to their reduced solvent capacity, SCTs may allow the precipitation of bioactive agents. However, MCTs have increased portal vein absorption through the liver, whereas LCTs have increased lymphatic absorption and they are not subject to the first-pass effect [20]. When selecting an emulsifier system, it is also important to consider chemical stability and lipase inhibition [21]. Thus, nonionic and ionic surfactants are used to coat the oil-water interface of lipid nanoparticles. Cremophors and Pluronics are the most common emulsifiers used in lipid-based nanoparticles. The addition of cationic or anionic surfactants into lipid nanoparticles can increase the charge of the particulate surface and prevent electrostatic aggregation. The storage stability of lipid nanoparticles must also be considered, and the induction of GI irritation is an important safety issue when administering ionic surfactants. The coating of the emulsifiers with high surface charge or steric structure can avoid the aggregation between the nanoparticles. It is generally recognized that the zeta potential of >30 mV or <−30 mV is preferred to maintain a long-term stability for nanoparticles. The decoration of polyethylene glycol on lipid nanoparticle surface is well known to demonstrate the repulsion between particles via steric hindrance. The GI tract with harsh environment also causes the instability of nanocarriers. The strategies of increasing surface charge and steric repulsion are also useful to increase the stability in the GI environment. In particular, some surfactants increase the viscosity of the nanosystem, leading to increased bioadhesiveness and prolonging residence time in the GI tract [22].

Inclusion of bioactive agents using lipid nanocarriers is a major technique that is used for oral delivery of nutrient-grade ingredients, such as vitamins, antioxidants, and fatty acids [23]. Vitamins are essential organic micronutrients, many of which function as enzyme cofactors. Many people worldwide take dietary supplements with vitamins to prevent obesity, cardiovascular disorders, osteoporosis, skin aging, and various cancers. Vitamins can be classified as water-soluble or lipid-soluble. The water soluble vitamins are B1 (thiamine), B2 (riboflavin), B3 (niacin), B5 (pantothenic acid), B6 (pyridoxal), B7 (biotin), B9 (folic acid), and B12 (cyanocobalamin), and C. The fat-soluble vitamins are A (retinol, retinal, and retinyl esters), D2 (ergocalciferol), D3 (cholecalciferol), E (tocopherol), K1 (phylloquinone), and K2 (menaquinone). In addition, these vitamins have many derivatives or analogs that have different bioactivities. The oral bioavailability of most vitamins is relatively low because of their low bioaccessibility, chemical instability, and poor GI absorption [24]. In addition to their low bioavailability, oral vitamins may have erratic absorption profiles, high intra- and inter-subject variations, and absorption that is not dose-dependent, all of which complicate oral administration. However, research in nanomedicine has led to the development of lipid nanocarriers that may improve the bioavailability of oral vitamins.

The objective of this review is to outline the pharmacokinetics of oral vitamins that are formulated using lipid-based nanoparticles. We focus on studies that used different lipid nanocarriers to encapsulate vitamins, such as SEDDSs, nanoemulsions, microemulsions, SLNs, and NLCs. Most of the lipid nanocarriers that we discuss are nanosystems based on oil-in-water (*o*/*w*). We do not consider liposomes and niosomes, because these molecules are nanovesicles with aqueous cores and lipid shells, rather than nanoparticles with a lipid matrix. We conclude with a discussion of the emerging applications of vitamin-loaded lipid nanoparticles.

## 2. Oral Delivery of Vitamins

Vitamins are organic micronutrients that are essential to the human body and important for the maintenance of normal metabolism, cellular regulation, growth, and development. Humans require 13 dietary vitamins [25]. For people at risk of vitamin deficiency, an oral supplement is generally the first treatment [26]. However, some vitamins have low oral bioavailability due to degradation, poor GI transport, and low water solubility. Thus, it is essential to develop novel forms of oral vitamins to improve absorption.

### 2.1. Vitamin A

**Figure 3:**
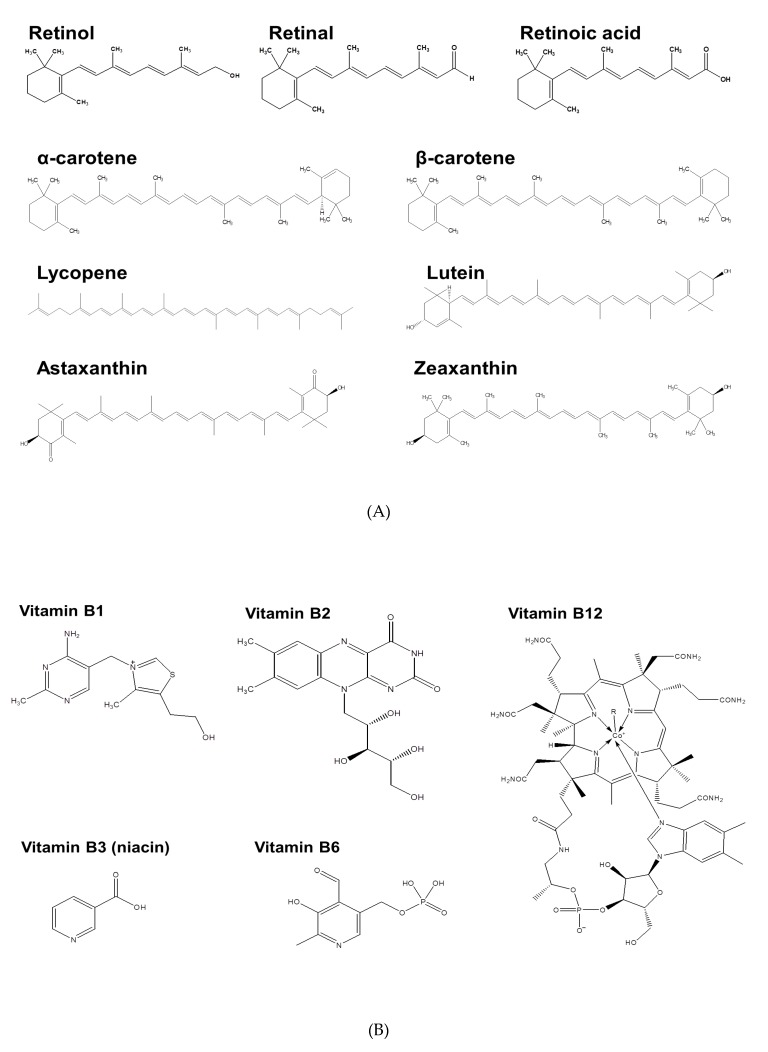

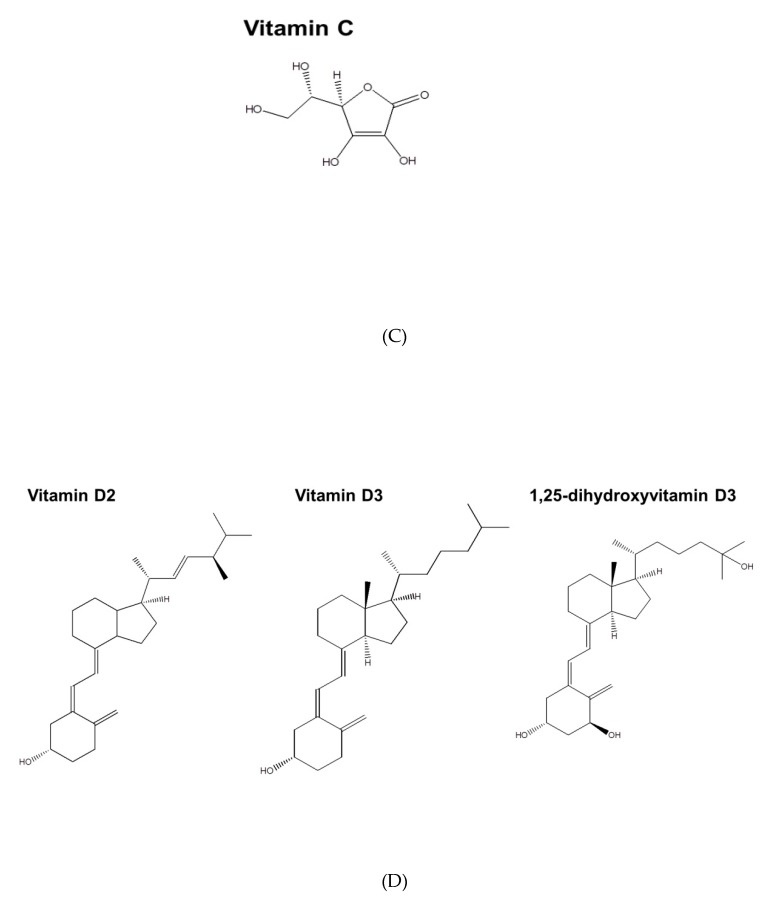

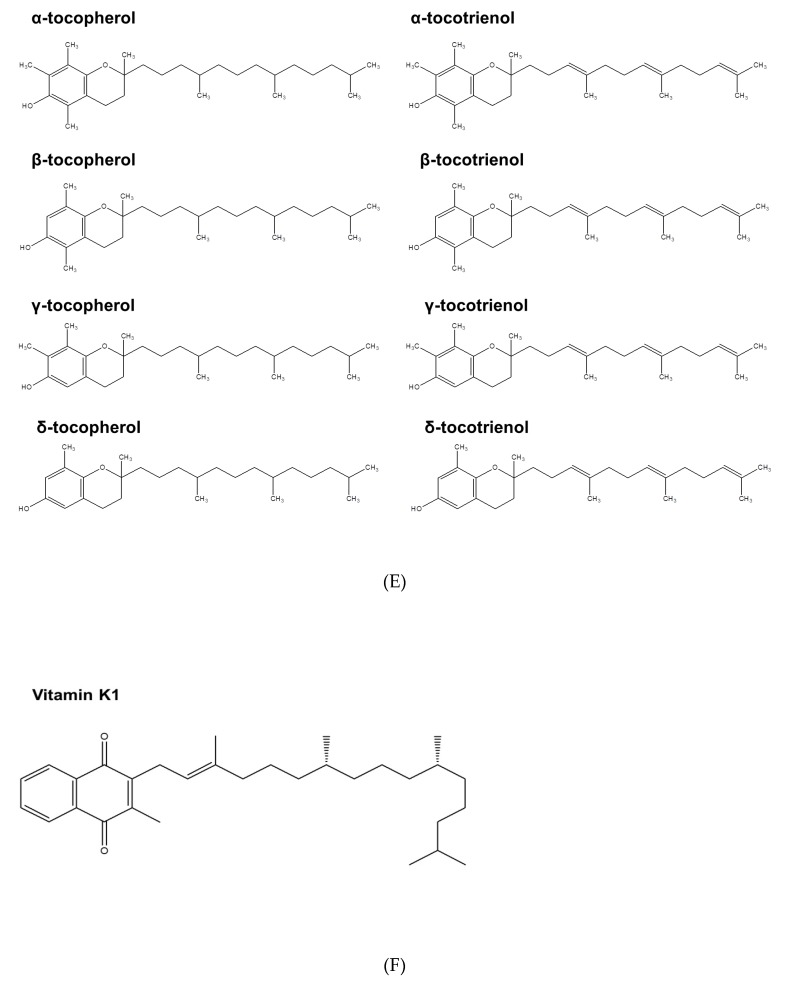
Chemical structures of (**A**) vitamin A; (**B**) vitamin B; (**C**) vitamin C; (**D**) vitamin D; (**E**) vitamin E; and, (**F**) vitamin K.

Fat-soluble vitamins have important roles in the synthesis and degradation of nutrients, immune function, homeostasis, and growth [27]. The A vitamins are unsaturated fat-soluble organic compounds, including retinol, retinal, retinoic acid, and some provitamin A carotenoids (Figure 3A). They have important roles in vision, reproduction, bone growth, and function of the immune system and skin. Oral carotenoids, which are bioconverted to vitamin A, are often recommended for disease prevention. In particular, epidemiological studies reported that the consumption of carotenoid-rich foods is associated with decreased risk of several cancers, cardiovascular diseases, macular degeneration, and cataracts [28]. β-carotene is the most commonly used carotenoid in functional foods and pharmaceutical products, because of its strong provitamin A and antioxidant activities.

The current recommended dietary allowance (RDA) of vitamin A is 600 μg retinol activity equivalents (RAE) per day for adult females and 800 μg for adult males [29]. The dose, absorption efficiency, and percentage of bioconversion of provitamin A into vitamin A impact the circulation level after intake [30]. Thus, total oral intake should consist of approximately 65% vitamin A and about 35% carotenoids. The use of vitamin A and carotenoids in the food industry is limited, due to their low oral bioavailability, poor solubility, and chemical instability. It is believed that vitamin A transfers from foods or oral doses into lipid droplets (dietary fat emulsions) that are present in the GI lumen during ingestion. About 70% of vitamin A is stored in the liver, and the liver uptakes vitamin A chylomicron remnants through cell surface receptors, such as the low density lipoprotein (LDL) receptor, LDL receptor-related protein 1 (LRP1), and heparan sulfate proteoglycans (HSPGs) [31]. It is likely that chylomicron-remnant retinyl esters and β-carotene are released from hepatocytes during chylomicron remnant metabolism. Several human genetic variations can modulate vitamin A and provitamin A bioavailability and metabolism.

### 2.2. Vitamin B

Vitamins B and C are water-soluble molecules that function as cofactors for many enzymes. The vitamin B group consists of B1, B2, B3, B5, B6, B7, B9, B12, and related derivatives (Figure 3B). Among these, B1, B2, B3, B6, and B12 play important roles in disease prevention. For example, vitamin B1 is a cofactor of dehydrogenase and transketolase; polyneuritis, Alzheimer’s disease, and colon cancer are linked to B1 deficiency [27]. Vitamin B2 is a precursor to flavin adenine dinucleotide (FAD) and flavin mononucleotide (FMN), which are cofactors for flavoenzymes, a large group of oxidoreductases. B2 deficiency can cause mucosal disorders, skin disorders, and anemia. Vitamin B3 is the precursor of nicotinamide adenine dinucleotide (NAD) and nicotinamide adenine dinucleotide phosphate (NADP), and B3 deficiency is associated with pellagra, depression, and dementia. Vitamin B6 is a cofactor for enzymes that function in the metabolism of amino acids, carbohydrates, and lipids [32]. The monitoring of B6 concentration is important for patients with cardiovascular diseases. Vitamin B12 has a complex structure, with a corrin ring and an embedded cobalt ion. This vitamin is important for erythrocyte formation, nerve cell maintenance, and DNA synthesis. B12 deficiency may lead to megaloblastic anemia. Dairy products are the major dietary sources of vitamin B12, although this vitamin has bioavailability of 8 to 12% from milk preparations [33], but 12 to 33% following the consumption of tofu and cheese [34].

### 2.3. Vitamin C

Vitamin C is the only water-soluble vitamin not in the vitamin B group (Figure 3C). It is one of the most essential vitamins and it has roles in many physiological processes, including immune response and iron absorption [35]. Vitamin C is abundant in many fruits and vegetables, such as mango, kiwi fruit, papaya, lettuce, tomato, and strawberry. Vitamin C is also a strong antioxidant that can reduce oxidative stress. Vitamin C may improve physiological function by increasing baroreflex sensitivity, ameliorating vascular conductance, decreasing systemic inflammation, reducing cancer cell-specific toxicity, and augmenting the inotropic response to β-adrenergic stimulation [36]. Oral administration is always less effective than intravenous administration because of the low bioavailability of this vitamin [37]. Sodium-dependent vitamin C transporters 1 and 2 are important for intestinal vitamin C absorption and renal reabsorption. Sodium-dependent vitamin C transporter 2 also promotes the entry of vitamin C into the most metabolically active tissues and cells [38]. Thus, the rate of intestinal absorption and renal reabsorption and excretion greatly impact the bioavailability of vitamin C.

### 2.4. Vitamin D

Vitamin D has two major forms: D2 and D3 (Figure 3D), each of which the body converts into the bioactive calcitriol (25-dihydroxyvitamin D). Ultraviolet irradiation of ergosterol in plants leads to the formation of vitamin D2, and ultraviolet radiation of 7-dehydrocholesterol in human skin leads to the formation of vitamin D3. Vitamin D is also obtained from foods, including egg yolk, fish, and milk. Vitamin D has important roles in the mineralization of bone and teeth, due to its regulation of calcium and phosphorus homeostasis [39]. There is also evidence that vitamin D supplements can prevent malignancies, cardiovascular diseases, osteoporosis, and diabetes.

Oral vitamin D absorption requires scavenger receptor class B type I (SR-BI), N-terminal Niemann-Pick C1 (NPC1), like intracellular cholesterol transporter 1 (NPC1L1), and cluster of differentiation (CD36) [40]. These three trans-membrane proteins primarily function as cholesterol transporters in the intestine. Although use of oral vitamin D supplements is a low-cost and practical method to treat deficiency, clinical advancements of vitamin D administration are limited by its lipophilic character, low solubility in GI fluid, and low bioavailability [41]. Moreover, the facile degradation of vitamin D by light, air, and heat limits the practicability of oral vitamin D. Thus, there is an urgent need to develop new formulations of vitamin D.

### 2.5. Vitamin E

Molecules in the vitamin E group, which function as antioxidants and free radical scavengers, include four tocopherols (α, β, γ, and δ) and four corresponding unsaturated tocotrienols (Figure 3E). These compounds have anti-inflammatory activities and they are recommended for the treatment of cardiovascular disorders and cancers [42]. In general, α-tocopherol has greater bioactivity than β- and γ-tocopherol (15 to 30% of α-tocopherol activity), and δ-tocopherol has very little bioactivity. Among the tocotrienols, only the α and β forms appear to have significant activity [43]. Oral absorption of vitamin E is considered to occur by passive transport across the enterocyte apical membrane [44], predominantly in the distal region of the small intestine (distal jejunum and ileum). The three vitamin D transporters (SR-BI, NPC1L1, and CD36) also function in vitamin E absorption. Vitamin E is also absorbed through the lymphatic system, where it diffuses as lipoprotein complex [45]. In particular, the lipoprotein complex that is formed by mixing vitamin E, bile salt micelles, and chylomicron is too large to pass across blood capillary, and it is only transported via the lymphatic system.

In United States, the RDA for vitamin E is 15 mg, but more than 90% of the population does not consume this amount [46]. The poor aqueous solubility and limited intestinal permeability of vitamin E account for its limited bioavailability [47]. In addition, vitamin E is highly susceptible to oxidation when exposed in oxygen, light, and heat. Thus, the oral bioavailability of tocopherol and tocotrienols ranges from 10 to 33% [44,48]. Notably, α-tocopherol also competes for GI absorption with other fat-soluble vitamins, such as γ-tocopherol, and vitamins A, D, and K [49]. An increasing number of investigations have attempted to increase vitamin E bioavailability by the use of nanoparticles. Encapsulation of vitamin E within nanoparticles can impede its interactions with other fat-soluble vitamins, which would otherwise inhibit vitamin E absorption.

### 2.6. Vitamin K

Vitamin K is essential for the activation of specific proteins that function in bone metabolism and blood clotting. Based on its source, vitamin K is classified as plant-derived vitamin K1 (phylloquinone) or animal/bacteria-derived vitamin K2 (menaquinones) (Figure 3F). Vitamin K1 is a procoagulant that is used in cases of hemorrhage, and vitamin K2 has roles in the regulation of blood clotting factors, namely prothrombin and five other proteins (Factors VII, IX, and X, and proteins C and S) [50]. During the process of pancreatic lipolysis, vitamin K is solubilized in mixed micelles with bile salts, and is then absorbed through the proximal intestine. Vitamin K is insoluble in GI fluid, and a low blood level of vitamin K and its metabolites is often due to low dietary intake [51]. Following oral administration of vitamin K, there are large inter- and intrasubject variations in absorption, and because of this, its bioavailability can range from 10 to 63% [52].

## 3. Lipid Nanoparticles

**Figure 4:**
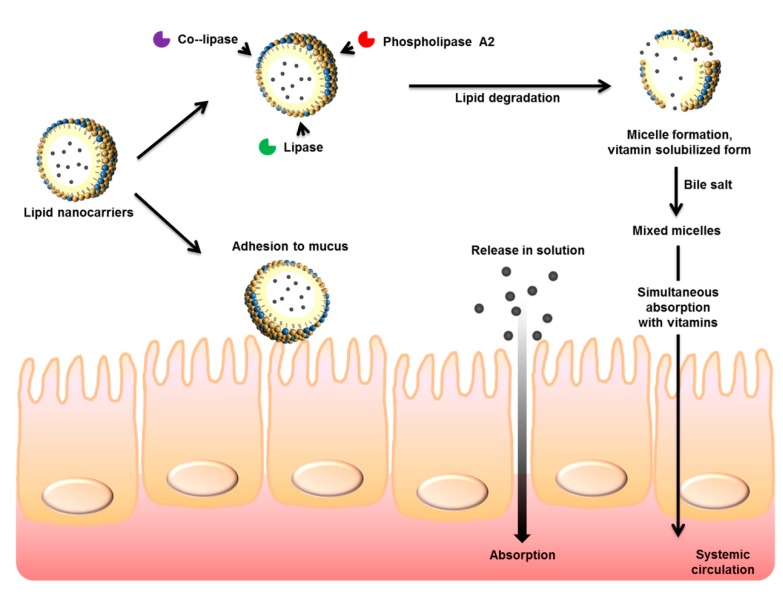
Possible pathways of gastrointestinal absorption of orally administered lipid nanoparticles.

Lipid-based nanodelivery systems, such as SEDDSs, nanoemulsions, microemulsions, SLNs, and NLCs, have great promise as oral vehicles for the delivery of bioactive agents because they can increase the solubility and improve bioavailability. Thus, many researchers have examined the effect of lipid nanocarriers on pharmacological or bioactive efficacy, adverse effects that are associated with conventional formulations, and compliance by patients and consumers. Orally administered lipid nanoparticles can be absorbed by several different mechanisms (Figure 4). The ingredients of lipid nanoparticles include bioactive compounds, lipids, surfactants, aqueous solvents, and cosolvents, and the excipients are usually biocompatible and less toxic to the human body [53]. It is generally easy to scale up the production of liquid nanoparticles, and this is a major benefit when the product is to be used for commercial or clinical purposes.

### 3.1. Self-Emulsifying Drug Delivery Systems (SEDDSs)

**Figure 5:**
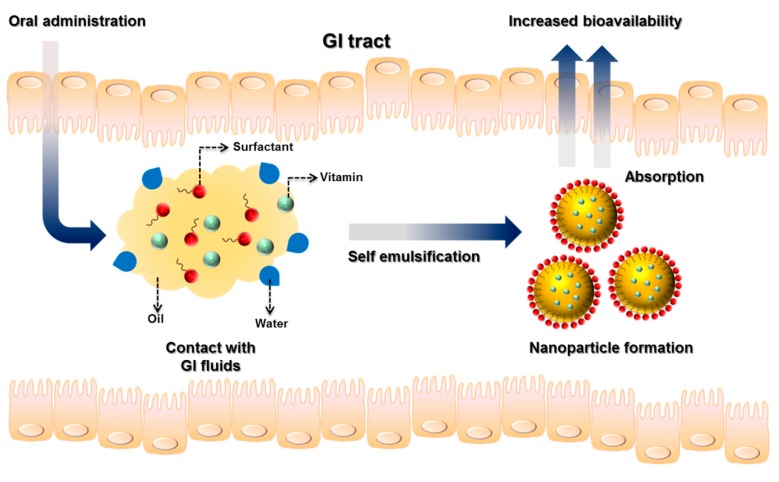
Formation of self-emulsifying drug delivery systems (SEDDSs) in the gastrointestinal tract.

SEDDSs are the most commonly employed lipid nanocarriers that are used to enhance the oral absorption of vitamins. A SEDDS consists of an anhydrous isotropic mixture of oil, emulsifier, coemulsifier, solubilizer, and active ingredient, in which spontaneously created *o*/*w* nanoemulsions or microemulsions with diameters below 300 nm form upon dilution with water under gentle agitation. Their unique ability of self-assembly in the GI fluid makes the drug or nutrient available as nano-sized oil droplets, and the high interfacial surface area improves dissolution in the GI environment (Figure 5) [54].

The two types of SEDDs are self-nanoemulsifying drug delivery systems (SNEDDSs) and self-microemulsifying drug delivery systems (SMEDDSs). SNEDDSs are generally opaque or translucent, and the droplet diameter is below 100 nm; SMEDDSs form transparent microemulsions of thermodynamically stable systems after oral ingestion [55]. The ingredients, droplet diameter, digestibility of the lipids, and lipophilicity of the bioactive agents all affect the bioavailability of an agent from SEDDSs. The selection of suitable excipients is vital for SEDDS preparation and for increasing bioavailability. SEDDSs are generally designed using the lipids and surfactants that are generally recognized as safe (GRAS). The methods that are employed for fabrication of SEDDSs include low-energy emulsification, the phase inversion temperature method, the phase inversion composition method, and solvent displacement [56].

Large volumes of conventional emulsions must be consumed to assure the therapeutic absorption of the bioactive agents because of the need for water in these emulsions. This large amount of water may increase hydrolysis and precipitation during long-term storage, thus reducing stability and oral absorption. SEDDSs are delivery systems of emulsion preconcentrates, and no water is used in their formulation. The concerns regarding conventional emulsions can be resolved with good patient compliance. When SEDDSs are used for oral dosage, the advantages are improved physicochemical stability, a possibility of filling capsules with the vehicle, and increased patient acceptability [57]. Digestive motility provides most of the agitation that is needed for forming nanoemulsions in the GI tract. The process of self-assembly requires no input of free energy (ΔGº) for the creation of nanoemulsions, so their development is thermodynamically spontaneous. In particular, the liquid crystalline phase formed between the oil and water phases swells, allowing for the spontaneous formation of an interface between the oil droplets and external phase [58]. Ingestion of SEDDSs increases the total amount of lipids in the GI tract, and these lipids can induce secretion of bile into the lumen. The increased bile salts, phospholipids, and cholesterol in the presence of lipids and emulsifiers provide a lipid-rich environment that favors the production of emulsion droplets. Thus, poorly soluble drugs or bioactive compounds that are initially dissolved in SEDDSs partition into the micelles. Micelle formation is an important step for increasing solubilization and absorption.

When considering oral administration, SEDDSs increase bioavailability by increasing the stability of bioactive agents in the GI environment because first-pass effects are minimal, they have increased lymphatic transport, and they inhibit P-gp-mediated efflux [59]. Some additional advantages of SEDDSs are that they can be easily delivered by the oral route, intersubject variability and effects of food are reduced, there is a fast onset of action, lower doses can be used, and they are easy to manufacture [56]. However, a disadvantage of SEDDSs is that they must be delivered through soft or hard gelatin capsules. The material in the capsule shell might be incompatible with the excipients, leading to precipitation of the active ingredients, the need for storage at low temperatures, and the use of specific preparation methods [60]. This problem can be resolved by transforming liquid SEDDSs into the solid state. Thus, techniques, such as freeze drying, spray drying, granulation, and adsorption to carriers, can be used to produce more stable and convenient forms for handling and delivery.

In addition to their roles as bioactive agents in SEDDSs, some vitamins and their derivatives can be used as excipients in the nanocarriers to assist absorption [61]. P-gp-mediated efflux is primarily responsible for the low bioavailability of some drugs and actives. Vitamin E and d-α-tocopheryl polyethylene glycol succinate (TPGS) appear to counteract the effects of P-gp. In particular, vitamin E can suppress P-gp activity and TPGS is used as a surfactant in lipid nanoparticles to enhance intestinal transport due to its potent anti-P-gp activity [62].

### 3.2. Nanoemulsions

Nanoemulsions are heterogeneous mixtures of oil droplets in an aqueous medium that are stabilized by an emulsifier system. The emulsified mixture is isotropic and translucent and it is kinetically stable, in that there is no flocculation or coalescence during long-term storage. In contrast to SEDDSs, nanoemulsions are produced by directed assembly rather than self-assembly. Drugs can be loaded inside the oil cores of nanoemulsions before administration to improve absorption when administered orally. The United States Food and Drug Administration has approved some nanoemulsions of poorly soluble drugs, such as Estrasorb^®^, Flexogan^®^, and Restasis^®^ [63]. In addition to drugs, nanoemulsions can also encapsulate nutrients and vitamins, and they have several advantages over food systems; these advantages include the ability to incorporate lipophilic entities, high physical stability, easy modulation of product texture, and rapid GI digestibility [64].

Generally, nanoemulsions are prepared from components that are GRAS or natural products. To reduce toxicity and increase stability, surfactants or cosurfactants are used in nanoemulsions, such as peptides (peptide surfactant AM1), proteins (caseinate and whey protein isolate), polysaccharides (Arabic gum and modified starch), phospholipids (egg lecithin and soybean lecithin), and small molecule nonionic surfactants (Span and Tween) [23,65]. High pressure homogenization is the major technique that is used to produce nanoemulsions. The use of rapidly diffusing, electrostatically stabilized, and low molecular weight emulsifiers can provide complete dispersivity. The other techniques used to fabricate the stable nanoemulsions are the use of a microfluidizer, a sonicator, and low energy approaches [66]. Due to their tiny droplet size and large surface area, they easily interact with the biological components of the GI tract, and are therefore efficient carriers for bioactive compounds because they provide better oral absorption than conventional emulsions. Nanoemulsions reportedly increase bioavailability because they increase solubilization, prolong gastric residence time, stimulate lymphatic absorption, reduce the effects of efflux transporters, and inhibit metabolism [67]. Vitamins, especially fat-soluble vitamins, can be loaded within the oil core of liquid droplets, where they are protected from chemical and enzymatic degradation and released after ingestion.

### 3.3. Microemulsions

Conventional emulsions are optically opaque or turbid because the coarse droplets have diameters that are similar to the wavelength of light (hundreds of nanometers), so they scatter light strongly. Nanoemulsions can be regarded as conventional emulsions with droplets of 10 to 100 nm in diameter. Conventional (coarse) emulsions and nanoemulsions are thermodynamically unstable, because the separated oil and aqueous phases have a lower total free energy than the emulsified oil and water [66]. However, microemulsions are thermodynamically stable systems, whose free energy is lower than that of the phase-separated components, so they tend to form spontaneously or with a small input of energy [68]. Table 1 compares the properties of these three types of emulsions.

Microemulsions are generally optically transparent and they can be spherical, ellipsoidal, or worm-like in shape, depending on the molecular geometry of the surfactants. The different domains of microemulsions can be characterized by ternary-phase diagrams. Three basic components—two immiscible liquids and a surfactant—are needed to produce micoemulsions. Most microemulsions use oil and water as immiscible liquid pairs. The relative amounts of these three components can be represented in a ternary phase diagram. Stable and transparent microemulsions only form in a specific region of the ternary phase diagram. Relative to nanoemulsions, a large amount of emulsifiers is necessary to prepare stable microemulsions.

The physicochemical characteristics and thermodynamic stability of microemulsions allow for spontaneous formation by low energy emulsification, known as the titration method. The benefits of orally applied microemulsions for drug and nutrient delivery are improved solubilization, protection from degradation, and increased GI transport [81]. The large content of surfactants in microemulsions increases GI membrane fluidity and subsequent permeability [82]. Neoral^®^ is an example of an oral microemulsion formulation containing cyclosporine A that was approved by the United States FDA. Microemulsions are also used for solubilization and improving the bioavailability of nutraceuticals and vitamin derivatives, such as coenzyme Q10, lutein, and carotenoids [83].

### 3.4. Solid Lipid Nanoparticles (SLNs)

The pharmaceutical and food industries are devoting increasing attention to SLNs, because they are not subject to many of the deficiencies of microcapsules and conventional colloidal carrier systems [84]. SLNs are composed of melt-emulsified lipids that are solid at room temperature and in the body. Thus, they are colloidal nanosystems consisting of crystalline lipids. Because of the unique features of their particulate structure, SLNs have the advantages of controlled release, increased bioavailability, improved stability, and suitability for industrial-scale production [85]. The avoidance of organic solvents and the use of biocompatible and biodegradable solid lipids in their preparation increase their applicability and safety. Natural and synthetic solid lipids are added into SLNs to form stable nanosystems, including triglycerides, glyceryl monostearate, glyceryl behenate, glyceryl palmitostearate, wax, fatty acids, and cholesterol [86]. SLNs can be manufactured using melt homogenization, cold homogenization, or melt microemulsification [5]. Hot homogenization is the most-used method, because the solid lipids must be melted to the liquid phase to facilitate mixing with other components in the production of small particles. Cold homogenization may be used for drugs or bioactive agents that are sensitive to heat.

Digestion of oral SLNs starts in the stomach with the action of gastric lipases. The mechanical mixing of gastric fluid with SLNs leads to a crude emulsion [87]. Intestinal fluids then further digest SLNs. The small size of SLN particles allows them to adhere to GI mucus and enter the intervillar space. The emulsifiers that coat the surface of SLNs allow increased absorption, because they reduce membrane fluidity. The solid state of the lipid nanoparticle matrix protects chemically labile bioactive agents and prolongs release of the drug or vitamin. It is also possible to load SLNs into capsules or pellets for more convenient application [69].

### 3.5. Nanostructured Lipid Carriers (NLCs)

Despite the advantages of SLNs for oral delivery of bioactive agents, several drawbacks must be resolved before their application. Due to the densely packed solid lipid matrix, there is only a small loading space for the bioactive agent. Moreover, particulate aggregation and gelation may occur during storage, and this can lead to the expulsion of the drug or bioactive agent from the nanoparticle. The “burst escape” of drugs for some oral SLNs can increase their toxicity. Thus, further research is required to improve the formulations used for SLNs. The incorporation of a liquid lipid into the crystalline matrix can increase imperfection (lattice defects) in the core, thereby increasing entrapment of the active ingredients [70].

NLCs are second-generation lipid nanoparticles composed of a mixture of liquid and solid lipids. These carriers have improved physical stability. Moreover, the release of bioactive agents from NLCs can be easily modulated by adjusting the ratio of liquid and solid lipids.

A number of preparation platforms are suitable for the production of NLCs. These include high pressure homogenization, solvent evaporation, emulsification-solvent diffusion, solvent injection, phase inversion, microemulsion, multiple emulsion, sonication, and membrane extrusion [88]. High pressure homogenization is preferred over other methods because this method has matured over many years of use in the pharmaceutical industry, and because it does not require a solvent.

NLCs are especially useful for enhancing GI absorption by lymphatic uptake via M cells and because first-pass effects can be disregarded. NLCs increase carrier transport through the stagnant layer (between the intestinal bulk fluid and brush border of enterocytes) and thereby promote absorption [71]. Surfactants on the shells of NLCs also inhibit P-gp efflux. Certain lipophilic drugs can be loaded into NLCs to improve GI transport. Research has shown that NLCs improve the bioavailability of etoposide by 3.5-fold, iloperidone by 8.3-fold, silymarin by 2.5-fold, and tamoxifen by 2.7-fold, relative to control suspensions or commercial products [72]. NLCs are also used as carriers to increase the bioavailability of water-insoluble nutrients, such as coenzyme Q10 [89].

## 4. Enhancement of Oral Bioavailability of Vitamins Using Lipid Nanocarriers

Vitamin deficiencies can adversely affect human health and even cause certain diseases. Despite significant efforts during the past few decades, the prevention and treatment of vitamin deficiency remains far from satisfactory. Conventional formulations of oral vitamins usually have insufficient bioavailability and often have adverse effects. Lipid nanocarriers can resolve many of these problems for vitamins that are administered by the oral route. In particular, lipid nanocarriers allow for modulation of the size, surface charge, ingredients, and targeting of specific ligands, and they also have greater solubility, stability, and bioactivity.

### 4.1. Self-Emulsifying Drug Delivery Systems (SEDDSs) for Oral Vitamin Delivery

SEDDSs are the most widely used lipid nanoparticles used to improve the oral absorption of vitamins. The self-assembly of nanosystems makes them attractive for the engineering of nanomedicines with distinct physicochemical properties, and greatly simplifies the optimization of formulations. Previous research examined the use of SEDDSs for oral delivery of five nutraceuticals with poor water solubility (vitamin A, vitamin K2, coenzyme Q10, resveratrol, and quercetin) [73]. The researchers optimized the formulations to fill gelatin capsules. A dispersion test indicated that all formulations containing nutrients dispersed spontaneously to form microemulsions, with droplet diameter of 25 to 200 nm. For example, vitamin K2-loaded nanocarriers had an average diameter of about 40 nm. Thus, the development of such formulations is a feasible approach to improve the oral absorption of nutraceuticals with low solubility.

SEDDSs are the preferred formulation for incorporation into capsules or tablets for oral ingestion. There is also interest in transforming liquid SEDDSs into solid forms. Thus, previous research fabricated vitamin A SEDDSs, consisting of soybean oil, Cremophor EL, and Capmul MCM-C8, and mixed them with microcrystalline cellulose (Avicel^®^) to generate solid powders that can be compressed into tablets [74]. The researchers then orally administered these tablets (vitamin A dose: 7.5 mg/kg) to rats for pharmacokinetic examination. The average peak concentration (C_max_) was higher for the SEDDS tablets (656 ng/mL) than the conventional tablets (421 ng/mL), indicating improved oral bioavailability.

Lutein is a naturally occurring carotenoid that can prevent cataracts and age-related macular degeneration, but it has poor water solubility. The reported oral bioavailability of lutein is only 5%, and there are large inter-individual variations [90]. Sato et al. [91] developed solid SEDDSs with lutein (droplet diameter: 337 nm) to increase its transport into the lymphatic system. They conducted thoracic lymph cannulation in rats to measure lymphatic lutein concentrations. The lutein concentration for the control powder was about 100 ng/mL at 9 h, but it was 250 ng/mL for the SEDDSs. This indicates that SEDDSs increased lutein diffusion from the intestine into the lymph stream. Shanmugan et al. [75] prepared SEDDSs containing phosphatidylcholine as the oil phase to promote oral lutein absorption by spray-drying liquid SEDDSs to form a solid powder. These SEDDSs had a droplet diameter of 92 nm and a polydispersity index (PDI) of 0.208. The lutein dissolution from SEDDSs was 79% within 30 min. Analysis of the pharmacokinetic parameters following oral administration to rabbits at a lutein dose of 5 mg/kg indicated that the SEDDSs had C_max_ values that were about 21-fold greater than lutein powder, and eight-fold greater than the commercial product.

Based on the literature [76], MCTs are preferred over LCTs for SEDDSs because they have higher fluidity, better solubilization, and the ability to self-emulsify. Grove et al. [77] compared the pharmacokinetics of two SEDDSs, one containing MCT and the other containing LCT, for oral delivery of seocalcitol (a vitamin D analog) by giving rats 47 μg/kg of oral ^3^H-seocalcitol. The bioavailability was 45% for the MCT-SEDDSs and 18% for the LCT-SEDDSs, thus confirming the superiority of MCTs for use in the oil phase.

Vitamin E can be absorbed via lymph, where it is transported as a lipoprotein complex, and SEDDSs are ideal carriers for vitamin E because they enhance lymphatic absorption. Thus, a pharmacokinetic study in eight healthy humans compared vitamin E-loaded SEDDSs (400 IU of α-tocopherol) with commercial capsules (Natopherol^®^) [78]. The SEDDSs had a 2.2-fold greater bioavailability than the capsules (control). The SEDDSs also had a lag time of 2.1 h, shorter than that of capsules (5.0 h). These results indicated that SEDDSs provided more rapid delivery of α-tocopherol.

Tocotrienols, compounds in the vitamin E family that have a chroman head and a 16-C phytyl chain, have some bioactivities that are not present in the tocopherols, including lowering of cholesterol, suppression of tumors, and neuroprotection [92]. However, the oral bioavailability of tocotrienols is low due to their poor GI absorption. In addition, the biological half-life (t_1/2_) of tocotrienols is four- to five-fold shorter than α-tocopherol [79]. Yap and Yuen [80] produced two different tocotrienol-loaded SEDDSs that were easily lipolyzed under in vitro conditions, and they had a finer dispersion with negligible lipolysis. A single dose bioavailability test in six healthy subjects indicated that the area-under-curve (AUC) of each SEDDS was two to three times greater than a soybean oil solution. The two nanosystems also had comparable absorption and faster onset of GI absorption.

Supplementary vitamin E can slow the progression of atherosclerosis. Thus, Rasool et al. [93] prepared tocotrienol-loaded SEDDSs and conducted a randomized, placebo-controlled, clinical study to evaluate arterial compliance and vitamin E blood level. They assessed arterial compliance using carotid femoral pulse wave velocity and the augmentation index. The results indicated a linear dose response with circulatory tocotrienol concentration for oral doses of 50, 100, and 200 mg. In addition, the SEDDSs provided greater plasma tocotrienol level than the placebo. The SEDDSs also had improved arterial compliance within two months of treatment.

Alqahtani et al. [94] compared the oral bioavailability of γ-tocotrienol-SEDDS and δ- tocotrienol-SEDDS with commercial capsules (Unique E^®^). The nanocarriers had a mean diameter of 211 nm and a mean PDI of 0.5. Measurement of cellular uptake by human epithelial colorectal adenocarcinoma (Caco-2) cells indicated that the SEDDSs provided three-fold greater tocotrienol permeability than the commercial product (control). Moreover, SEDDSs significantly increased the bioavailability of the γ and δ forms in rats. Relative to the capsules, the AUC of δ-tocotrienol-SEDDS was seven-fold greater for 0.5 mg/kg doses and three-fold greater for 2.5 mg/kg doses. Nevertheless, this effect was self-limiting, because a corresponding increase in the free surfactant level negatively impacted tocotrienol transport via NPC1L1. Alqahtani et al. [95] compared the lipolysis and oral bioavailability of γ-tocotrienol in SEDDSs to Tocovid^®^, a soft gelatin capsule formulation that is used to enhance tocotrienol absorption. The nanocarriers had a diameter of 117 nm and a PDI of 0.5. Analysis of in vitro lipolysis indicated the SEDDS provided a two-fold increased γ-tocotrienol solubilization. They also determined the plasma γ-tocotrienol level following oral administration to rats. The SEDDSs had a two-fold greater bioavailability at 10, 25, and 50 mg/kg doses.

Other researchers developed a polyethylene glycol conjugate of an α-tocopherol isomer (TPGS) that was water-soluble to be used for treating low birth weight infants and cholestasis [96]. Because of the surfactant, TPGS can self-assemble to form lipid nanoparticles. Another study examined the oral absorption of TPGS-SEDDSs with molecular weights of 350 Da (diameter: 10.8 nm) and 1000 Da (diameter: 61.6 nm) in rats [97] Following oral dosing at 1 mg/kg, the absolute bioavailability of TPGS350 was 18.2% and that of TPGS1000 was 16.6%; these values were three-fold higher than that of γ-tocotrienol in SEDDSs.

Other researchers encapsulated vitamin K1 into SEDDSs to develop lyophilized tables for and examined oral delivery [98]. They prepared these SEDDSs using Labrasol and Transcutol as the excipients. The droplet diameters size of a series of SEDDSs was 92 to 263 nm, depending on the mixture. An in vitro release study indicated that the commercial tablets released 28% of vitamin K1 after 1 h; the optimized SEDDSs tablets released 99% of vitamin K, which is due to the smallest droplet size of 82 nm. Analysis of the pharmacokinetics of vitamin K1 from SEDDSs and commercial tablets in human volunteers indicated that SEDDSs increased vitamin K1 absorption, with a bioavailability that was 170% of that provided by the commercial products. The AUC of these oral SEDDSs was similar to that obtained from intramuscular injection. Table 1 summarizes the profiles of SEDDSs formulations used for oral vitamin delivery.

Vitamin E and its derivatives can also function as additives, rather than the active ingredient, in lipid nanocarriers, because vitamin E is a strong antioxidant that can stabilize oxygen-sensitive drugs or bioactive agents in lipid nanosystems [61,99]. TPGS is a non-ionic surfactant that can prevent drug precipitation and promote supersauration in lipid nanosystems [100]; it can also interact with drugs by hydrogen bonding, thereby accelerating drug dissolution [101]. Previous research reported that TPGS could inhibit presystemic drug metabolism and intestinal efflux mediated by P-pg, and increase oral drug bioavailability [62,102]. Yang et al. [103] evaluated the oral pharmacokinetics of paclitaxel (an anti-cancer agent) in SEDDSs with TPGS1000 as a P-gp inhibitor. Because of the high affinity for P-gp and the first-pass effect by cytochrome P450 enzymes, oral administration of paclitaxel is not very effective [104]. An aqueous dilution of paclitaxel-loaded SEDDSs led to droplet diameters of only 2 nm. Oral administration of these SEDDSs to rats at 2, 5, and 10 mg/kg indicated that the SEDDSs increased the bioavailability by 29% to 53% (depending on the dose) relative to commercial Taxol^®^. This result suggests that TPGS have great potential for increasing drug absorption. Other research examined paclitaxel-loaded SEDDSs in patients with advanced cancers to determine the oral efficiency [105]. The results indicated that TPGS1000 increased paclitaxel solubility. In these experiments, all of the patients received 160 mg on days 1, 8, and 15. The AUC was similar for oral SEDDSs (2.06 μg∙h mL^−1^) and Taxol^®^ (1.97 μg∙h mL^−1^). However, oral SEDDSs had a shorter t_max_ (2 h) than Taxol^®^ (4 h), suggesting that TPGS increased the absorption rate of paclitaxel.

Docetaxel is a second-generation of taxoid with greater anticancer potency than paclitaxel. Valicherla et al. [106] developed docetaxel-loaded SEDDSs containing TPGS1000 (droplet diameter: 160 to 180 nm), and evaluated oral absorption and therapeutic activity. The SEDDSs had a 25-fold increased cytotoxicity against breast cancer (MCF-7) cells relative to the free drug. The absolute bioavailability of SEDDSs in rats (22%) was higher than that of Taxotere^®^ (7%). The chylomicron flow blocking and tissue distribution indicated the importance of lymphatic transport of SEDDSs, and that this accounted for its greater tumor accumulation than Taxotere^®^.

Cyclosporine A is a lipophilic cyclic polypeptide used as an immunosuppressant drug for immunomodulation in transplant recipients. Oral dosing has limited effectiveness, because of its limited aqueous solubility, low intestinal permeation, and P-g efflux [107]. Thus, researchers incorporated TPGS1000 in SEDDSs as an efflux inhibitor to increase the intestinal absorption of oral cyclosporine A [108]. The optimized nanocarriers had good stability in simulated GI fluids. These SEDDSs had faster drug release and 4.5-fold greater oral bioavailability than the commercial product (Bioral^®^). The nephrotoxicity of these SEDDSs was negligible, based on measurements of blood urea nitrogen and plasma creatinine.

Sirolimus (rapamycin) is a triene macrolide with potent immunosuppressive effects due to its inhibition of T-cell activation. However, the poor water solubility and low stability of this drug limit its use as an oral agent. Cho et al. [109] designed solid SEDDSs (mean droplet diameter: 108 nm) for sirolimus to improve its dissolution, stability, and GI absorption. Based on surfactant screening, TPGS1000 was a more effective stabilizer in simulated gastric fluid than Poloxamer, Gelucire, and Sucroester. The addition of TPGS1000 also increased the t_1/2_ of sirolimus from 5.2 to 100.3 min. These researchers also administered oral sirolimus to rats at a dose of 5 mg/kg for the analysis of its pharmacokinetics. Relative to the commercial Rapamune^®^ solution, the SEDDS had a greater AUC (363 vs. 484 μg∙h mL^−1^) and a shorter t_max_ (2.4 vs. 1.0 h).

Cefpodoxime is an oral, third-generation cephalosporin antibiotic. Previous research attempted to improve its solubility, GI permeation, and oral bioavailability by use of SEDDSs, with TPGS and Tween 80 as surfactants and Capmul as the oil phase (average diameter: 55 to 60 nm) [110]. An in vitro permeability test, performed in a Franz diffusion cell using goat intestine as the diffusion barrier, indicated that the drug flux was much greater for the SEDDSs than the free control (0.985 vs. 0.104 μg/cm^2^/min). Administration of oral formulations to rats at a dose of 100 mg/kg indicated the SEDDSs provided a 5.4-fold increased AUC relative to the plain cefpodoxime.

Fenofibrate is a cholesterol-lowering drug with a high lipophilicity, whose oral bioavailability in hard gelatin capsules is about 60%, but highly variable among individuals [111]. Wei et al. [112] investigated the oral bioavailability of fenofibrate formulated with SEDDSs, consisting of TPGS1000 and Tween 20 or Tween 80 as surfactants. Fenofibrate release from the SEDDSs was complete within 30 min, but release from the commercial product (Tricor^®^) was limited. A pharmacokinetic study of healthy volunteers examined the effect of different forms of oral fenofibrate at a dose of 54 mg. Tricor^®^ had the greatest AUC (87 μg∙h mL^−1^), followed by SEDDSs containing Tween 20 (53 μg∙h mL^−1^) and SEDDSs containing Tween 80 (52 μg∙h mL^−1^). Consumption of water with oral SEDDSs and the agitation of GI tract may be insufficient to enhance the self-emulsifying process that is necessary for in vivo dissolution. Another study examined the oral absorption of SEDDSs (droplet diameter: 205 to 379 nm, depending on the oil:surfactant ratio) loaded with fenobribrate that were composed of Myritol (oil phase) and TPGS1000 (surfactant) [113]. These researchers then selected two optimized SEDDSs for analysis of the pharmacokinetics in healthy subjects receiving oral fenofibrate containing Tween 80 or with a high oil:surfactant ratio. The t_max_ was greatest for Tricor^®^ (2.8 h), followed by Tween 80 SEDDSs (2.0 h) and high-oil SEDDSs (1.8 h). The bioavailability was also 1.2-fold greater for high-oil SEDDSs than Tricor^®^. However, loading fenofibrate into Tween 80 SEDDSs reduced the AUC. Table 2 summarizes the pharmacokinetic parameters of oral drug delivery assisted by TPGS-containing SEDDSs that were designed to increase bioavailability.

### 4.2. Nanoemulsions/Microemulsions for Oral Vitamin Delivery

Nanoemulsions and microemulsions are other approaches used to improve the solubility and GI transport of vitamins [114]. Vitamins that are formulated in nanoemulsions have better physical stability in electrolyte dispersions and plasma [115]. The group of Dr. McClements (University of Massachusetts) performed a series of experiments to investigate the effect of different nanoemulsion formulations on the GI bioaccessibility of β-carotene (a provitamin A). More specifically, they examined the influence of the oil matrix of nanoemulsions on the GI diffusion of β-carotene [116]. They created nanoemulsions consisting of Tween 20 as an emulsifier, with LCTs, MCTs, or orange oil as the carrier oils, and then measured bioaccessibility across a GI membrane using an in vitro model to simulate the gastric and intestinal phases. The bioaccessibility significantly depended on the type of oil in the nanoemulsion. Thus, the bioaccessibility of β-carotene was near 0 in orange oil (due to an inability to form mixed micelles that solubilize β-carotene). However, the bioaccessibility was relatively high for LCTs (66%), but it was low for MCTs (2%), indicating that the mixed micelles with MCTs were too small to solubilize β-carotene.

The team of Dr. McClements also compared the bioaccessibility of β-carotene from predissolved nanoemulsions, a physical mixture with nanoemulsions, and phosphate buffered saline [117]. They constructed the *o*/*w* nanoemulsions using corn oil as the lipid core. Measurement of bioaccessibility using a simulated GI model indicated that the β-carotene crystals in PBS had the lowest transport, which they attributed to the small number of mixed micelles that are available for the solubilization of β-carotene. The solubilized β-carotene in nanoemulsions was transferred from the oil droplets into mixed micelles in the lipid digestion step, leading to a bioaccessibility of 69%. The interfacial layer surrounding the oil droplets in nanoemulsions is essential to maintain the long-term stability of nanosystems [118]. Thus, Liu et al. [119] utilized ternary conjugates of covalent polyphenol, protein, and carbohydrate cross-links to stabilize the oil drop surface of β-carotene-loaded nanoemulsions. The nanoparticles that were coated with surface-active chlorogenic acid-lactoferrin-polydextrose had better stability in terms of droplet fusion in the GI tract, and therefore increased β-carotene bioaccessibility. Their interpretation was that this was because the formation of a thick interfacial layer by the conjugates suppressed the ability of lipase to adsorb to the oil droplets. Mun et al. [120] incorporated β-carotene-loaded nanoemulsions into rice starch hydrogels and then determined the in vitro bioaccessibility in simulated GI tract. The oil droplets were stable in the simulated stomach, but they aggregated in the simulated small intestine. Moreover, when they added β-carotene into the nanoemulsions, the bioaccessibility increased from 1% to 23% due to the formation of mixed micelles in the simulated small intestine fluid. The further incorporation of hydrogels increased the bioaccessibility to about 50%, presumably because the hydrogel matrix protected the oil droplets from coalescence in the gastric phase.

*Rhinacanthus nasutus* is a medicinal herb rich in carotenoids that has anticancer activity [121]. Ho et al. [122] isolated the carotenoids from *R. nasutus* to prepare microemulsions with enhanced oral bioavailability in rats. The microemulsions were prepared using Capryol 90, Transcutol, and Tween 80, and the mean size was 10.4 nm. A carotenoid suspension or microemulsions were fed to rats at a dose of 20 mg/kg. The absolute bioavailability of carotenoids was 0.11% for the aqueous dispersion and 0.45% for the microemulsions.

Other researchers examined the anti-inflammatory effect of vitamin D-loaded nanoemulsions in ovalbumin-activated asthmatic mice [123]. The nanoemulsions contained Miglyol 812, Cremophor RH40, Tween 80, and Transcutol and the Balb/c mice received oral vitamin D at a dose of 2000 IU/kg. The nanoemulsions had a greater C_max_ (26.3 vs. 38.3 ng/mL) and a shorter t_max_ (5.22 vs. 3.56 h). Treatment with nanoemulsions also decreased the myeloperoxidase activity, O_2_^−^ level, and cytokine production, and attenuated asthma. Salvia-Trujillo et al. [124] evaluated the impact of nanoemulsion droplet size on the in vitro vitamin D2 bioaccessibility and in vivo absorption in humans. The nanoemulsions had mean droplet diameters of 112, 530, and 14,500 nm. The in vitro lipid digestion experiments indicated the complete digestion of the small- and medium-sized nanoemulsions, but not the large ones (83%). However, in vivo oral absorption was greatest for the largest emulsions. Differences between the simulated GI tract and the actual GI tract may partly explain the poor correlation of the in vitro and in vivo results.

Parthasarathi et al. [125] examined the impact of droplet size on vitamin E absorption by preparing nanoemulsions (diameter: 277 nm) with conventional emulsions (diameter: 1285 nm). The saponin-coated nanoemulsions had greater stability when subjected to heating, long-term storage, and mechanical stress. They also administered the vitamin E to rats at a dose of 100 mg/kg. The nanoemulsions had a greater C_max_ (11.6 vs. 2.6 μg/mL) and a shorter t_1/2_ (0.85 vs. 1.11 h). Natural vitamin E can be derived from crops (nuts, grains, and vegetables) and nanoemulsions can be an excellent system for the oral absorption of natural vitamin E [47]. Fabrication of vitamin E-loaded nanoemulsions led to an average diameter of 88 nm, and the AUC of vitamin E in nanoemulsions was 1.6-fold greater than commercial soft capsules in rats. The nanoemulsions were also stronger antioxidants than soft capsules in rats with oxidative damage due to d-Galactosamine.

As with SEDDSs, vitamin E derivatives may be used in nanoemulsions to improve oral drug absorption. Thus, previous researchers used nanoemulsions containing TPGS1000 with paclitaxel to enhance oral absorption in rats [126]. These nanoemulsions had a diameter of 21.6 nm and a PDI of 0.13. Upon oral administration, the commercial product (Taxol) had plasma below 300 ng/mL and a bioavailability of 10.6%. Paclitaxel from nanoemulsions was rapidly absorbed, and it had a C_max_ of 3.5 μg/mL within 30 min and a bioavailability of 70.6%.

Acetylpuerarin is a promising agent for the amelioration of coronary heart disorder and arrhythmia, because it decreases myocardial oxygen consumption [127]. Sun et al. [128] tried to develop nanoemulsions to enhance oral acetylpuerarin delivery and to improve its therapeutic potential. Their nanoemulsions had an average diameter of 150 nm and were stabilized by TPGS1000 with acetylpuerarin encapsulation. The AUC of oral acetylpuerarin nanoemulsions in rats was 5.76 μg∙h mL^−1^; this value was 2.6-fold greater than the suspension and 1.7-fold greater than the oil solution. They also used the chylomicron flow-blocking rat model to examine the role of lymphatic transport. The results showed a lower AUC in the blocking model than the control model, indicating the importance of lymphatic absorption for oral nanoemulsions. Table 3 shows the profiles for oral vitamin delivery by nanoemulsions and microemulsions.

### 4.3. SLNs for Oral Vitamin Delivery

Some investigations have examined the use of SLNs as oral delivery systems for vitamins and their analogs because they are biocompatible with the lipid matrix (consisting of triglycerides, fatty acids, or glycerol esters) and are readily degraded in vivo. Astaxanthin is a naturally occurring carotenoid that is abundant in salmon, shellfish, and shrimp, which has activity against several cancers and cardiovascular disorders, with greater potency than vitamin E and β-carotene [129]. However, its poor water solubility, light sensitivity, and decomposition in the presence of oxygen have limited its use in oral formulations. Thus, researchers have entrapped astaxanthin into SLNs composed of glycerol esters and Tween 20 [130]. These SLNs had a mean diameter of 163 to 167 nm and the encapsulation percentage was approximately 89%. The results showed that SLNs provided prolonged astaxanthin release in simulated GI juices.

The saturable process of intestinal γ-tocotrienol absorption (mediated by NPC1L1) leads to an oral bioavailability of only 9% [131]. Thus, researchers have also examined the use of SLNs to increase γ-tocotrienol permeation and bioavailability in human liver (HepG2) cells [132]. The results indicated that the SLN provided a two-fold increased uptake relative to mixed micelles. In addition, an in situ perfusion study, in which rats were given oral γ-tocotrienol (10 mg/kg) as SLNs or as mixed micelles, demonstrated that SLNs provided a 10-fold greater permeability. The AUC for SLNs was 12.1 μg∙h mL^−1^ and that for mixed micelles was 3.9 μg∙h mL^−1^.

Other researchers, in an attempt to increase the bioavailability of docetaxel (an anticancer agent), compared TPGS 1000-coated docetaxel-loaded SLNs (diameter: 189 nm) with Tween 80-coated docetaxel-loaded SLNs (diameter: 215 nm) [133]. The SLNs provided a more sustained release of docetaxel than the marketed product Taxotere^®^. In particular, after oral delivery, the AUC was 12.9 μg∙min/mL for TPGS-coated SLNs 7.0 μg∙min/mL for Tween 80-coated LNs, but only 3.9 μg∙min/mL for Taxotere^®^. This result confirmed that TPGS inhibited docetaxel efflux.

Other researchers used TPGS 1000-coated SLNs with curcumin to increase its GI absorption [134]. Curcumin is a natural compound that has an antioxidant, anti-inflammatory, chemopreventative, and anti-viral properties [135], but its clinical use is limited due to its low aqueous solubility, instability in GI fluid, and rapid elimination [136]. Thus, the application of *N*-trimethyl chitosan to the SLN surface formed an acid-resistant shell, which increased the stability. An oral curcumin suspension (50 mg/kg) had a C_max_ of 0.24 μg/mL and an AUC of 0.27 μg∙h mL^−1^; however, oral SLNs with curcumin had a C_max_ of 1.21 μg/mL and an AUC of 6.23 μg∙h mL^−1^. SLNs also improved curcumin targeting in the rat brain. Ji et al. [137] formulated curcumin into SLNs coated with TPGS and Brij^®^ 78. The optimized SLNs had an average diameter of 135 nm with 91% curcumin encapsulation. These SLNs exhibited no “burst release”, suggesting homogeneous curcumin distribution in the lipid core. An in situ GI absorption test, in which a curcumin suspension and SLNs were dosed at 50 mg/kg, found that SLN loading increased curcumin permeability across jejunum from 0.81 × 10^−4^ to 1.56 × 10^−4^ cm/s. The AUC of SLNs was 12.3-fold higher than the suspension.

### 4.4. NLCs for Oral Vitamin Delivery

It is also feasible to use NLCs as carriers to improve the oral absorption of lipid-soluble vitamins [89]. For example, previous researchers used hot pressure homogenization to develop NLCs that entrapped the lipophilic vitamin D3 [138]. These NLCs were stable and had a mean diameter of 133 nm. The diameter remained unchanged in simulated gastric fluid, but it increased to 216 nm in simulated intestinal fluid. An in vitro digestion test demonstrated that NLCs provided controlled release of vitamin D3, in that they were not degraded in simulated stomach fluid, but they released more than 90% of the vitamin D3 into intestinal fluid.

Dacarbazine is a highly lipophilic and light-sensitive anticancer drug with very low oral absorption [139]. Almousallam et al. [140] fabricated NLCs that were coated with TPGS to improve dacarbazine absorption. These NLCs encapsulated about 99% of this drug and the diameter of the nanoformulations was 155 nm. The in vitro release of the drug was biphasic, with 50% released during the first 2 h and sustained release for up to 30 h. Other researchers used TPGS-coated NLCs to encapsulate sulforaphane, a natural anti-cancer agent derived from broccoli, cauliflower, and cabbage, to improve oral bioavalaibility [141]. Sulforaphane has documented effectiveness against pancreatic, breast, hepatic, melanoma, and prostate malignancies [142]. The particle diameter was 145 nm and the encapsulation efficiency was 85%. Measurements of intestinal transport in vitro showed that NLC loading increased the permeability coefficient from 0.67 × 10^−4^ to 4.82 × 10^−4^ cm/min. In addition, examination of the in vivo pharmacokinetics in rats indicated that NLCs provided a five-fold enhancement of oral bioavailability relative to the control suspension. The sustained release of sulforaphane from NLCs also increased the t_max_ from 2.5 to 7.5 h.

## 5. Conclusions

When designing different formulations to improve the bioavailability of an oral vitamin, it is essential that the carrier stabilizes the vitamin and improves its transport into circulation. This review summarized recent advances in the use of vitamin-loaded lipid-based nanocarriers that were designed to enhance oral bioavailability. The selection of the carrier is important, and it should ideally provide maximal activity and minimal side effects. The use of lipid nanoparticles has numerous advantages over conventional formulations for dosing of vitamins, because they are more stable, they can provide sustained release, they can target different tissues, and they provide increased bioavailability. Some important limitations of conventional formulations, such as low solubility and poor epithelium permeation, can also be resolved by the use of lipid nanocarriers. Self-assembled lipid nanoparticles are frequently utilized to improve the oral delivery of vitamins. The type of emulsifier, particle size, interfacial composition, and vitamin concentration are the major factors that impact oral absorption. The comparison of different lipid-based nanoparticles used for enhancing oral vitamin delivery is summarized in Table 4. Our introduction and description of the lipid-based nanocarriers that are used for vitamin delivery provide an overview for investigators who are attempting to design feasible and efficient delivery systems for vitamins and other bioactive agents. In the near future, it may be possible to extend the use of lipid nanoparticles by using them as vehicles for other functional nutrients. 

## Figures and Tables

**Table 1:** Characterization of self-emulsifying drug delivery systems (SEDDSs) loaded with vitamins or their derivatives and their oral absorption.

Vitamin	Average Size	Model Animals	Outcomes Offered by Nanoparticles	Reference
Vitamins A and K2	25–200 nm	None	Good dispersity to form microemulsions	Shah et al. [69]
Vitamin A	Unknown	Rat	An increased bioavailability of 1.4-fold compared to control	Taha et al. [70]
Lutein	337 nm	Thoracic lymph-cannulated rat	An increased bioavailability of 2.5-fold compared to control	Sato et al. [71]
Lutein	92 nm	Rabbit	An increased bioavailability of 11.8-fold compared to control	Shanmugam et al. [72]
Seocalcitol	29 nm	Rat	A 45% relative bioavailability was achieved	Grove et al. [73]
α-tocopherol	Unknown	Human	An increased bioavailability of 2.2-fold compared to commercial capsules	Julianto et al. [74]
Tocotrienols	1.5–10.6 μm	Human	An increased bioavailability of 2–3-fold compared to control	Yap and Yuen [75]
Tocotrienols	Unknown	Human	Improvement of arterial compliance and oral bioavailability compared to placebo	Rasool et al. [76]
Tocotrienols	211 nm	Rat	An increased bioavailability of 3–7-fold compared to commercial capsules	Alqahtani et al. [77]
γ-tocotrienol	117 nm	Fed rat	An increased bioavailability of 2-fold compared to commercial capsules	Alqahtani et al. [78]
TPGS350 and TPGS1000	11–62 nm	Rat	An increased bioavailability of 3-fold compared to γ-tocotrienol SEDDSs	Abu-Fayyad et al. [79]
Vitamin K1	82–263 nm	Human	An increased bioavailability of 1.7-fold compared to commercial tablets	El-Say et al. [80]

TPGS, d-α-tocopheryl polyethylene glycol succinate.

**Table 2:** Characterization of self-emulsifying drug delivery systems (SEDDSs) incorporated with d-α-tocopheryl polyethylene glycol succinate (TPGS) as the surfactants for enhancing the oral absorption of drugs.

Active Ingredient	Average Size	Model Animals	Outcomes Offered by Nanoparticles	Reference
Paclitaxel	2 nm	Rat	An increased bioavailability of 1.3–1.5-fold compared to Taxol^®^	Yang et al. [97]
Paclitaxel	Unknown	Patients with cancers	A decreased t_max_ of 2-fold compared to Taxol^®^	Veltkamp et al. [99]
Docetaxel	160–180 nm	Rat	An increased bioavailability of 3.2-fold compared to Taxotere^®^	Valicherla et al. [100]
Cyclosporine A	72 nm	Rat	An increased bioavailability of 4.5-fold compared to Bioral^®^	Jain et al. [102]
Sirolimus	108 nm	Rat	An increased bioavailability of 1.3-fold compared to Rapamune^®^	Cho et al. [103]
Cefpodoxime	55–60 nm	Rat	An increased bioavailability of 5.4-fold compared to plain drug	Bajaj et al. [104]
Fenofibrate	Unknown	Human	The bioavailability was reduced by SEDDSs	Wei et al. [106]
Fenofibrate	205–379 nm	Human	An increased bioavailability of 1.2-fold compared to Tricor^®^	Lin et al. [107]

TPGS, d-α-tocopheryl polyethylene glycol succinate.

**Table 3:** Characterization of nanoemulsions and microemulsions loaded with vitamins or their derivatives and their oral absorption.

Vitamin	Average Size	In Vitro or In Vivo Model	Outcomes Offered by Nanoparticles	Reference
β-carotene	140~170 nm	In vitro bioaccessibility	Increased bioaccessibility in simulatedGI environment (66%)	Qian et al. [110]
β-carotene	About 200 nm	In vitro bioaccessibility	Increased bioaccessibility in simulated GI environment (69%)	Xia et al. [111]
β-carotene	About 400 nm	In vitro bioaccessibility	Increased β-carotene stability and bioaccessibility in simulated GI environment	Liu et al. [113]
β-carotene	260 nm	In vitro bioaccessibility	Increased bioaccessibility in simulated GI environment (about 50%)	Mun et al. [114]
Carotenoids	10.4 nm	In vivo bioavailability in rat	An increased bioavailability of 4-fold compared to aqueous suspension	Ho et al. [116]
Vitamin D	Unknown	In vivo bioavailability in mouse	An increased bioavailability of 1.3-fold with asthma attenuation	Tang et al. [117]
Vitamin D2	112, 530, and 14500 nm	In vitro bioaccessibility and in vivo bioavailability	Increased bioavailability following the increase of droplet size	Salvia-Trujillo et al. [118]
Vitamin E	227 nm	In vivo bioavailability in rat	An increased bioavailability of 3-fold compared to conventional emulsions	Parthasarathi et al. [119]
Natural vitamin E	88 nm	In vivo bioavailability in rat	An increased bioavailability of 1.6-fold compared to soft capsules	Gong et al. [120]
TPGS as surfactant	21.6 nm	In vivo bioavailability in rat	An increased bioavailability of 6.7-fold compared to Taxol	Khandavilli and Panchagnula [121]
TPGS as surfactant	150 nm	In vivo bioavailability in rat	An increased bioavailability of 2.6-fold compared to aqueous suspension	Sun et al. [123]

TPGS, d-α-tocopheryl polyethylene glycol succinate.

**Table 4:** The comparison of different lipid nanocarriers for enhancing oral vitamin delivery.

Lipid Nanosystem	Nanoparticle Structure	Vitamins and Related Compounds Loaded
SEDDS	An anhydrous isotropic mixture of oil and emulsifier to spontaneously create nanoparticles in GI tract	Vitamin A, vitamin K1, vitamin K2, coenzyme Q10, lutein, and tocotrienols
Nanoemulsions/ microemulsions	The isotropic or heterogeneous mixtures to form oil droplets in an aqueous system stabilized by emulsifiers	Carotenoids, vitamin D, vitamin D2, and vitamin E
SLNs	The crystalline lipid structure in nanoparticles composed of melt-emulsfified lipids that are solid at room temperature	Astaxanthin and tocotrienols
NLCs	The second-generation lipid nanoparticles composed of a mixture of liquid and solid lipids for improving physical stability	Vitamin D3

SEDDS, self-emulsifying drug delivery systems; SLNs, solid lipid nanoparticles; NLCs, nanostructured lipid carriers; GI, gastrointestinal.
